# Supplementary material for: Trypanocidal Activity of Four Sesquiterpene Lactones Isolated from Asteraceae Species
Source: Molecules. 2020 Apr 25;25(9):2014. doi: 10.3390/molecules25092014 (PMC7248990; doi:10.3390/molecules25092014)

<u>EUPATORIOPICRIN ISOLATION</u>

S. maimarensis aerial parts (50 g)

↓ Maceration with dichloromethane (500 mL, 3 min., twice)

Crude dichloromethane extract

↓ Rotatory evaporator

Residue

↓ Taken with Ethanol-water (70:30) and then extracted with

i) Hexane (3 x 50 mL)

ii) Dichloromethane (3 x 50 mL)

Dewaxed dichloromethane extract

↓ Isocratic column chromatography on Si gel (230-400 Mesh) (Dichloromethane-ethyl acetate 1:2)

Column fractions

↓ TLC analysis:

_Silicagel 60 F_{254}

_Dichloromethane-ethyl acetate 1:2

_Anisaldehyde-sulfuric acid

Eupatoriopicrin rich fractions

↓ Solvent evaporation

Eupatoriopicrin precipitated as white crystals

↓ Washed with ethyl ether-ethyl acetate (7:3)

Eupatoriopicrin

90 mg

Purity: 94.6%

MINIMOLIDE ISOLATION

M. minima aerial parts (50 g)

↓ Maceration with dichloromethane (500 mL, 3 min., twice)

Crude dichloromethane extract

↓ Rotatory evaporator

Residue

↓ Isocratic column chromatography on Si gel (230-400 Mesh) (Dichloromethane-ethyl acetate 2:1)

Column fractions

↓ TLC analysis:
_Silicagel 60 F_{254}
_Dichloromethane-ethyl acetate 2:1
_Anisaldehyde-sulfuric acid

Minimolide rich fractions

↓ Solvent evaporation

Crystalline solid

↓ Recrystallization from heptane-ethyl acetate

Minimolide
250 mg
Purity: 99.8%

ESTAFIETIN ISOLATION

S. alpina aerial parts (50 g)

↓ Maceration with dichloromethane (500 mL, 3 min., twice)

Crude dichloromethane extract

↓ Rotatory evaporator

Residue

↓ Isocratic column chromatography on Si gel (230-400 Mesh) (Dichloromethane-ethyl acetate 9.5:0.5)

Column fractions

↓ TLC analysis:
_Silicagel 60 F_{254}
_Dichloromethane-ethyl acetate 9.5:0.5
_Anisaldehyde-sulfuric acid

Estafietin rich fractions

↓ Solvent evaporation

Solid residue

↓ Recrystallization from heptane-ethyl acetate

Estafietin
315 mg
Purity: 93.3%

EUPAHAKONENIN B ISOLATION

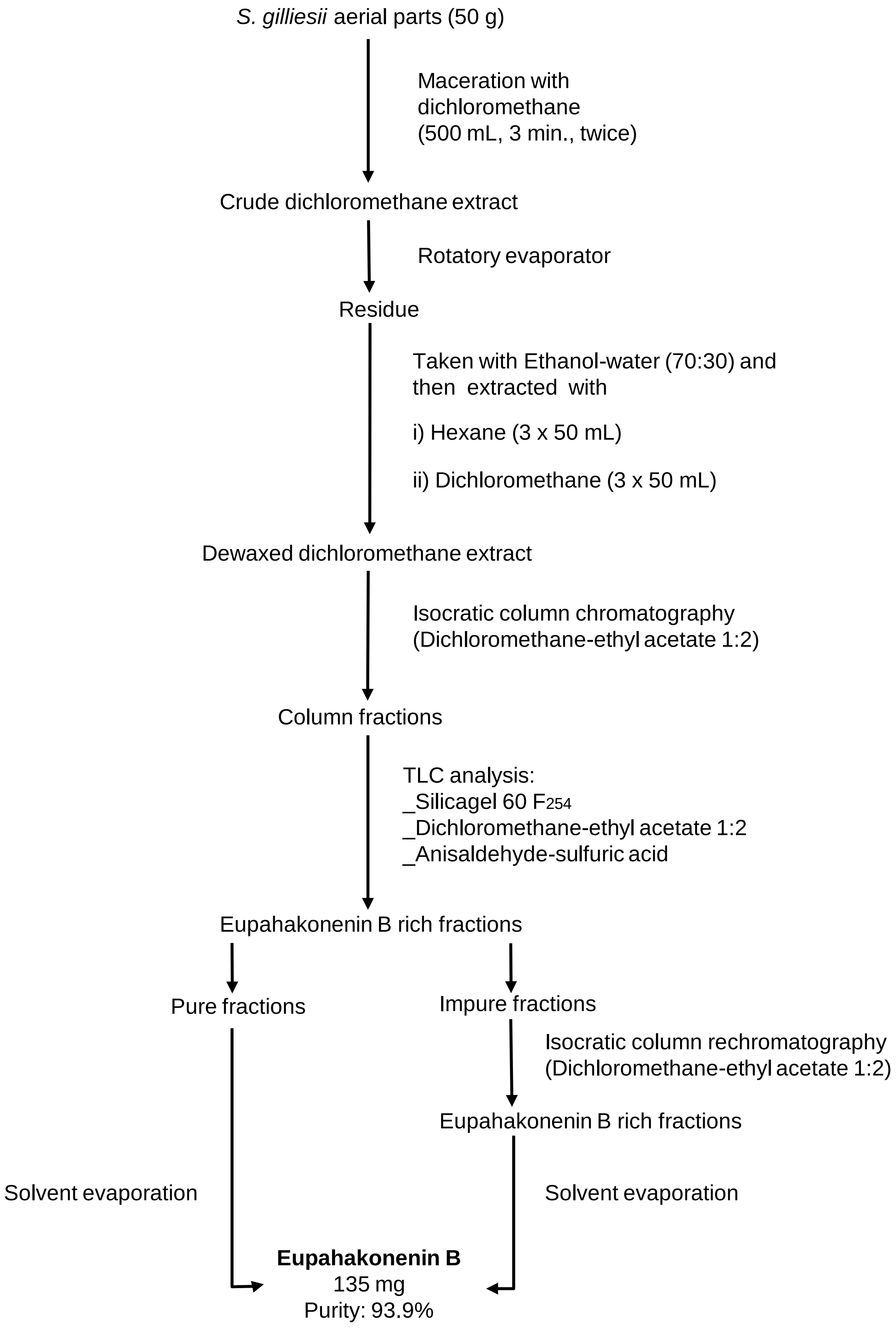

Supplement: Supplementary file 1 [file molecules-25-02014-s001.pdf]
